# Boric Acid Suppresses Cell Survival by Triggering Endoplasmic Reticulum Stress‐Induced Autophagy in Cervical Cancers

**DOI:** 10.1111/jcmm.70740

**Published:** 2025-07-19

**Authors:** Betul Keyif, Ceyhan Hacioglu

**Affiliations:** ^1^ Faculty of Medicine, Department of Gynecology and Obstetrics Düzce University Düzce Turkey; ^2^ Faculty of Medicine, Department of Medical Biochemistry Düzce University Düzce Turkey; ^3^ Faculty of Pharmacy, Department of Biochemistry Düzce University Düzce Turkey

**Keywords:** autophagy, boric acid, cervical cancer, endoplasmic reticulum stress

## Abstract

Cervical cancer ranks as the fourth most common cancer amongst women globally. This study aimed to investigate boric acid's effects on endoplasmic reticulum (ER) stress and autophagy signalling pathways in cervical cancer cells. We first assessed boric acid's effects on cell viability and proliferation in HUF and HeLa cell lines. Subsequently, we analysed cyclin D1 and CDK4 levels and boric acid‐induced nuclear morphology changes. We then examined autophagosome formation and mRNA expression of autophagy/ER stress markers (Beclin1, p62, LC3‐I/II, GRP78, p‐IRE1α, p‐PERK, CHOP and cleaved‐caspase‐3) in HeLa cells. The findings revealed that boric acid's IC50 was 3.17 mM for HUF cells but significantly lower (641.2 μM) for HeLa cells, indicating cancer cell sensitivity. In HeLa cells, boric acid‐induced a dose‐dependent decrease in cyclin D1 and CDK4 levels (associated with G1 phase arrest), which we did not observe in HUF cells. Additionally, boric acid treatment caused nuclear abnormalities in HeLa cells. Boric acid promoted autophagy by enhancing autophagosome formation and upregulating Beclin1, p62, and LC3‐I/II expression. Concurrently, it induced ER stress by increasing GRP78, p‐IRE1α, p‐PERK and CHOP expression. Furthermore, boric acid increased cleaved‐caspase‐3 expression and apoptotic cell counts. In conclusion, this study underscores boric acid's potential therapeutic effects in cervical cancer through ER stress and autophagy regulation.

## 1 Introduction

Cervical cancer ranks as the fourth most common malignancy affecting women globally, both in terms of incidence and mortality [1]. It is a prevalent and aggressive tumour of the female reproductive system, characterised by its high malignancy and rapid progression [2]. In 2018 alone, approximately 570,000 women were diagnosed with cervical cancer, and 311,000 succumbed to the disease worldwide [3]. While mortality rates vary significantly between countries, cervical cancer remains the leading cause of cancer‐related deaths amongst women in low‐income nations [4]. The primary etiological factor linked to cervical cancer is infection with the human papillomavirus (HPV) [5]. Currently, doctors primarily treat early‐stage cervical cancer through surgery; however, patients with high‐risk factors and those with intermediate to advanced stages typically require radiotherapy and chemotherapy [6]. Despite standardised treatment protocols, significant challenges remain in the management of cervical cancer. Notably, there is a pressing need to develop therapeutic strategies that minimise side effects and reduce harm to the human body.

The endoplasmic reticulum (ER) is a versatile organelle responsible for the biosynthesis of secretory, transmembrane and cytosolic proteins, as well as lipids [7]. To ensure proper protein folding, the ER contains high concentrations of chaperone proteins that assist in the correct folding of newly synthesised proteins [8]. Various cellular stressors can interfere with the ER's synthesis and post‐translational modifications [9]. Factors such as disruptions in the prooxidant/antioxidant balance, calcium misregulation, and impairments in protein folding or expression are strongly linked to carcinogenesis [10]. Consequently, protein folding capacity becomes compromised, leading to the accumulation of unfolded or misfolded proteins in the ER, a condition known as ER stress. ER stress has been closely associated with cancer progression, including invasion and metastasis [11]. Cells adapt to ER stress through the unfolded protein response (UPR), which is primarily activated by three signalling pathways: inositol‐requiring enzyme 1α (IRE1α), protein kinase R‐like ER kinase (PERK) and activating transcription factor 6 (ATF6). Under unstressed conditions, the chaperone glucose‐regulated protein‐78 (GRP78), also known as binding immunoglobulin protein (BIP), keeps these UPR pathways in check [12]. GRP78 binds to the hydrophobic regions of unfolded or misfolded proteins, helping to prevent their accumulation in the ER and restore normal ER function [13]. The activation of IRE1α begins when GRP78 dissociates, leading to the oligomerization and transphosphorylation of IRE1α, which then binds to unfolded proteins, further inducing ER stress. Similarly, GRP78's increased affinity for unfolded proteins triggers PERK activation. Once activated, PERK impacts essential biological processes such as amino acid synthesis and glycolysis [14]. In contrast, ATF6 activation occurs in the Golgi apparatus, where its proteolytic cleavage is crucial for driving the UPR [15]. In response to ER stress, the cell activates two key degradation mechanisms—proteasomal degradation and autophagy—as part of its adaptive response. The induction of UPR signalling significantly enhances autophagy, mainly through IRE1 and PERK pathways [16]. Like UPR, autophagy serves as an adaptive mechanism for tumour cells under environmental stress [17]. Autophagosomes initiate autophagy by sequestering damaged organelles and long‐lived proteins [18]. These autophagosomes then fuse with lysosomes, resulting in the degradation of their contents. Autophagy is intricately linked to the programmed cell death pathway, and excessive autophagy can induce cell death, known as autophagic cell death [19]. IRE1 and PERK signalling can elevate autophagic activity to the point where significant cellular contents, including key organelles, are degraded, ultimately leading to cell death [20].

Boron is a trace element found in various ecosystems such as soil, agglomerates and water. In nature, boron typically exists in compound forms, including borax, borates, and boric acid, rather than in its elemental state [21]. Due to its influence on redox balance and cellular metabolism, boron compounds have recently garnered significant attention for their effects on various signalling pathways, as explored in both in vitro and in vivo models [22]. Notably, boron compounds are known for their distinctive capacity to accumulate in tumour tissues, which has paved the way for the development of boron‐based compounds with potential applications in targeted cancer therapies [23]. In our prior studies, we demonstrated the therapeutic potential of boron‐containing compounds in treating different types of cancer cells and in vivo models, both for cancer treatment and the prevention of tissue damage in various pathophysiological conditions [24, 25]. Additionally, boron compounds, such as boric acid and borax, have shown promising effects on signalling pathways involved in autophagy, ferroptosis, and ferritinophagy, and have contributed to overcoming drug resistance in glioblastoma (GBM) treatment [26, 27]. Our previous research also indicated that borax can inhibit cell survival and proliferation in hepatocellular carcinoma cells by inducing ER stress [28]. Moreover, these compounds have produced encouraging results in preclinical studies and early‐phase clinical trials [29]. However, despite these promising findings, further research is necessary to fully understand the mechanisms behind the anticancer effects of boron‐containing compounds.

This study aimed to investigate the in vitro effects of boric acid on cervical cancer cells, specifically focusing on whether boric acid induces ER stress‐mediated autophagic cell death in HeLa cells. The study also explored the impact of boric acid on ER stress and autophagy‐related proteins to elucidate the underlying mechanisms.

## 2 Materials and Methods

### 2.1 Cell Culture and Treatments

Human primary uterine fibroblast (HUF) cells and human cervical adenocarcinoma (HeLa) cells were obtained from the American Type Culture Collection (ATCC). These cell lines were cultured in Dulbecco's modified eagle medium (DMEM; Gibco), supplemented with 10% foetal bovine serum (FBS; Sigma‐Aldrich), 100 units/mL penicillin, and 100 mg/mL streptomycin (Invitrogen). The cells were maintained in a humidified incubator set to 5% CO_2_ and 95% air at 37°C.

HUF and HeLa cell lines were cultured in medium without boric acid until they reached 80% confluency in an incubator maintained at 37°C with 5% CO_2_ overnight. Subsequently, the cells (2 × 10^5^ cells per well) were resuspended in 10% FBS DMEM and transferred into 96‐well culture plates. Afterward, the medium containing boric acid at concentrations of 100, 200, 400, 800 μM, 1.6 and 3.2 mM was added to the cells and incubated for 24, 48 and 72 h.

To investigate the effects of boric acid‐induced ER stress and induction of autophagy responses, 1 mM 4‐phenylbutyrate (4‐PBA) and 1 mM 3‐methyladenine (3‐MA) were applied to cells 2 h before boric acid treatment [30, 31].

### 2.2 Cell Viability Analysis

The MTT assay was employed to determine cell viability after treatment with boric acid at various concentrations (0–3.2 mM) for 24, 48 and 72 h. After incubation with 3‐(4,5‐dimethylthiazol‐2‐yl)‐2,5‐diphenyltetrazolium bromide (MTT) for 4 h at 37°C, dimethyl sulfoxide (DMSO) was added to dissolve the formazan crystals. The absorbance of the dissolved formazan was measured at 570 nm using a microplate reader (BioTek). The resulting absorbance values were used to assess the percentage of viable cells in each treatment group.

### 2.3 Cell Proliferation Analysis

Cell proliferation was measured using the BrdU incorporation method, which detects DNA synthesis in actively dividing cells. HUF and HeLa cells (2 × 10^5^ cells/well) were treated for 24 h with boric acid concentrations equivalent to the IC25 and IC50 values, as determined by the MTT assay. DNA synthesis in cells during the S phase was analysed by incorporating 5‐bromo‐2′‐deoxyuridine (BrdU). The BrdU incorporation was quantified using a BrdU detection kit (Sigma‐Aldrich, 2750) following the manufacturer's instructions, with absorbance measured at 450 nm. This provided an indication of the proliferative activity of the cells following boric acid treatment.

### 2.4 Nuclear Morphological Imaging

Nuclear morphology imaging was performed to observe structural changes in HeLa cell nuclei (2 × 10^5^ cells per well) following treatment with boric acid for 24 h. The cells were stained using 4′,6‐diamidino‐2‐phenylindole (DAPI; D8417, Sigma‐Aldrich), with incubation in DAPI solution for 30 min at 25°C in the dark. After incubation, the staining solution was discarded, and nuclear structures were examined via fluorescence microscopy. To enhance the visibility of nuclear details, counterstaining was applied.

### 2.5 Lactate Dehydrogenase Assay

To assess the cytotoxic effects of boric acid on HeLa cells, an LDH (lactate dehydrogenase) assay kit (E‐BC‐K046‐M) was used. In brief, HeLa cells were seeded at a density of 2 × 10^5^ cells per well in 12‐well plates and incubated with boric acid for 24 h. Following incubation, the cells were washed with phosphate‐buffered saline and then homogenised using an ultrasonic cell disruptor at 4°C. Cell debris was eliminated by centrifugation at 10,000 × *g* for 10 min at 4°C. The supernatant was collected, and LDH activity was measured at 450 nm following the manufacturer's protocol.

### 2.6 TUNEL Assay

The DeadEnd Colorimetric TUNEL System (G7130) was utilised for the in situ detection of apoptotic cells. This assay identifies apoptotic cells through the TdT (terminal deoxynucleotidyl transferase)‐mediated labelling of fragmented nuclear DNA ends. In this process, biotinylated nucleotides are added to the 3′‐OH ends of DNA fragments by TdT. Subsequently, horseradish peroxidase‐conjugated streptavidin (Streptavidin HRP) binds to the biotinylated nucleotides. The signal is then visualised using the chromogen diaminobenzidine (DAB), which produces a dark staining of apoptotic cell nuclei, allowing for observation under light microscopy (Oxion Inverso).

### 2.7 Autophagosome Detection Assays

HeLa cells were seeded in a 12‐well plate at a concentration of 2 × 10^5^ cells per well. Once the cells adhered to the plate and reached an appropriate density, they were treated with boric acid for 24 h. Following this, 100 μL of dansylcadaverine (DC, 30432 Sigma‐Aldrich) staining solution was added to each well. The cells were incubated at room temperature in the dark for 30 min, after which the staining solution was removed, and the cells were washed five times with wash buffer. Subsequently, 100 μL of collection buffer and one drop of anti‐quenching sealant were applied to cover the cell monolayer. The cells were then visualised using a fluorescence microscope equipped with a 488‐nm excitation filter and a 570‐nm emission filter, and images were captured accordingly. Additionally, DC positive cells (cells in which autophagy is induced) were analysed on the flow cytometry device via inducible dye at 488 nm using the CYTO‐ID autophagy detection kit, which allows the analysis of autophagic vacuoles.

### 2.8 Western Blot Analysis

HeLa cells were treated with boric acid for 24 h, then harvested with their culture medium and lysed in RIPA buffer at 4°C for 30 min. The lysate was subjected to sonication and then centrifuged at 10,000 × *g* at 10°C for 20 min. For SDS‐PAGE, 20 μg of total protein from each lysate was resolved on a 10% polyacrylamide gel and subsequently transferred onto a polyvinylidene difluoride (PVDF) membrane using the iBlot 2 Gel Transfer System (Thermo Fisher Scientific). Blocking, washing, and antibody incubation steps were carried out with the iBind Flex System (SLF2000; Thermo Fisher Scientific). Primary antibodies were applied to the membrane at optimised concentrations, followed by washing to remove excess. Secondary antibody incubation included HRP‐conjugated antibodies [HRP (A27036) at a 1:4000 dilution and Rabbit IgG H + L (A32731) at a 1:10000 dilution], which bind specifically to the primary antibodies used, namely: cyclin D1 (1:1000, PA5‐85257, Invitrogen), cyclin dependent kinase 4 (CDK4) (1:2500, PA5‐27827, Invitrogen), Beclin1 (1:1000, PA1‐16857, Invitrogen), LC3‐I/II (2 μg/mL, PA1‐16931, Invitrogen), p62 (1 μg/mL, P0067, Sigma‐Aldrich), GRP78 (2 μg/mL, PA1‐014A, Invitrogen), p‐PERK (1:1000, PA5‐40294, Invitrogen), p‐IRE1α (1:1000, PA1‐16927, Invitrogen), CHOP (1:1000, PA5‐104528, Invitrogen), and cleaved‐caspase3 (1:1000, PA5‐114687, Invitrogen). To visualise target proteins, a substrate was added, generating a detectable signal through reaction with the enzyme‐linked secondary antibodies. Band intensities were subsequently quantified using ImageJ software to measure protein expression levels.

### 2.9 Statistical Analysis

Data analysis and graphical visualisation were performed using GraphPad Prism 8. Experimental data were obtained from nine independent replicates, and the Shapiro–Wilk normality test was applied to assess normal data distribution. Results are presented as mean ± standard deviation (SD). For data following a normal distribution, differences in mean values were analysed using one‐way and two‐way ANOVA. Statistical significance was defined as *p* < 0.05.

## 3 Results

### 3.1 HUF and HeLa Cellular Viability Suppressed by Boric Acid

**FIGURE 1:**
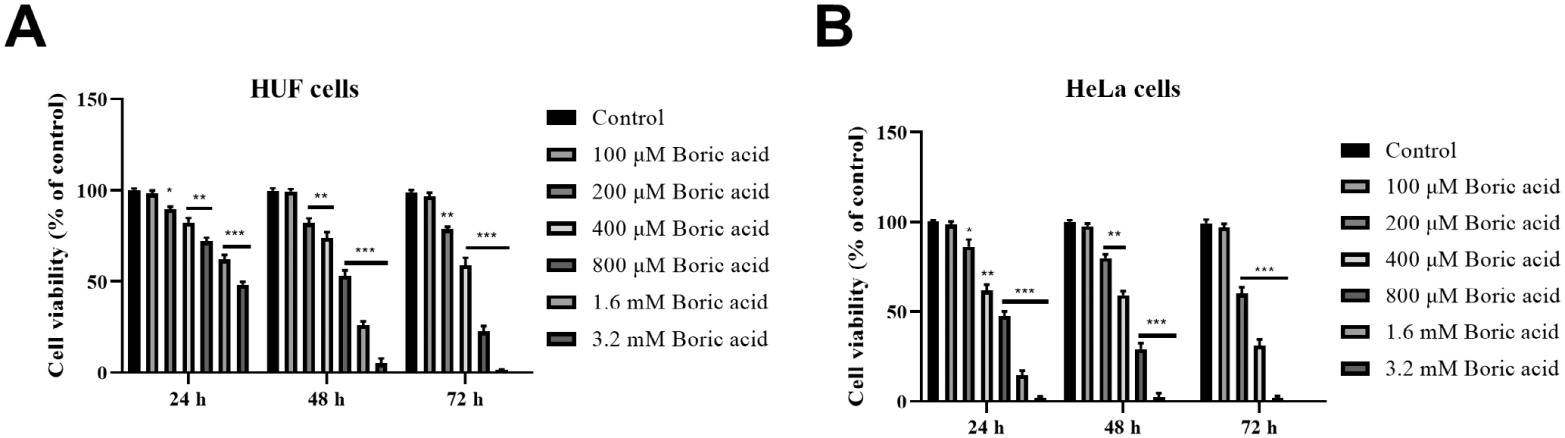
Effects of boric acid treatment on cell viability and proliferation of HUF and HeLa cells. (A) MTT results in HUF cells; (B) MTT results in HeLa cells. *p < 0.05, **p < 0.001, and ***p < 0.0001 vs. control groups.

According to the MTT assay results, boric acid treatment (0–3.2 mM) for 24, 48 and 72 h significantly reduced the viability of HUF cells (Figure 1A). However, treatment with 100 μM boric acid for 24, 48 and 72 h did not lead to a statistically significant decrease in the viability of HUF cells (*p* > 0.05 vs. control). After 24 h of treatment with 200, 400, 800 μM, 1.6 and 3.2 mM boric acid, HUF cell viability decreased by 10.5%, 17.4%, 27.2%, 34.6% and 51.1%, respectively, compared to the control group (*p* < 0.05, *p* < 0.001 and *p* < 0.0001). Similarly, 48‐h treatment with the same concentrations reduced HUF cell viability by 15.8%, 26.9%, 46.2%, 72.4% and 92.3%, respectively, compared to the control group (*p* < 0.001 and *p* < 0.0001). After 72 h, treatment with 200, 400, 800 μM and 1.6 mM boric acid led to a reduction in HUF cell viability by 21.2%, 40.5%, 74.9% and 98.2%, respectively, compared to the control group (*p* < 0.001 and *p* < 0.0001), while cell viability was completely abolished at 3.2 mM boric acid.

In HeLa cells, boric acid treatment also induced a time‐ and concentration‐dependent decrease in viability (Figure 1B). There was no statistically significant reduction in HeLa cell viability after 24 h of treatment with 100 μM boric acid (*p* > 0.05 vs. control). However, 24‐h treatment with 200, 400, 800 μM, 1.6 and 3.2 mM boric acid resulted in a 15.8%, 32.4%, 55.1%, 83.6% and 97.8% decrease in viability, respectively, compared to the control group (*p* < 0.05, *p* < 0.001 and *p* < 0.0001). After 48 h, treatment with 200, 400, 800 μM and 1.6 mM boric acid caused a reduction in HeLa cell viability by 19.5%, 37.5%, 79.5% and 98.1%, respectively, compared to the control group (*p* < 0.001 and *p* < 0.0001). At 72 h, 200, 400 and 800 μM boric acid reduced HeLa cell viability by 33.7%, 71.4% and 97.8%, respectively, compared to the control group (*p* < 0.0001). Notably, HeLa cell viability was completely abolished with 3.2 mM boric acid after 48 h and with 1.6 and 3.2 mM boric acid after 72 h. Overall, boric acid had a more pronounced effect on suppressing cell viability in HeLa cells compared to HUF cells. Based on MTT results, the IC50 for boric acid was determined to be 3.17 mM for HUF cells and 641.2 μM for HeLa cells. In subsequent experiments, HeLa cells were treated with 641.2 μM (IC50) and 350 μM (intermediate concentration) boric acid for 24 h.

### 3.2 Boric Acid Regulates Cell Proliferation and Cell Cycle in HUF and HeLa Cells

**FIGURE 2:**
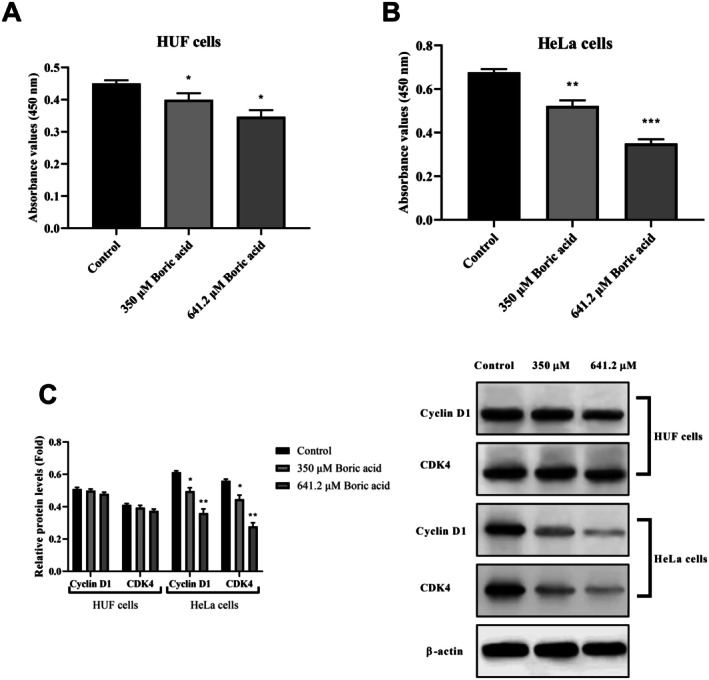
Effects of boric acid on cell proliferation and G1 phase‐related cell cycle proteins in HUF and HeLa cells. (A) BrdU incorporation in HUF cells; (B) BrdU incorporation in HeLa cells; (C) Cyclin D1 and CDK4 mRNA expressions in HUF and HeLa cells. *p < 0.05, **p < 0.001 and ***p < 0.0001 vs. control groups.

Consistent with the MTT results, the BrdU assay confirmed the antiproliferative effects of boric acid on both HUF and HeLa cells. Treatment with 350 and 641.2 μM boric acid for 24 h reduced HUF cell proliferation by 7.1% and 12.8%, respectively, compared to the control group (*p* < 0.05; Figure 2A). In contrast, boric acid exhibited a concentration‐dependent antiproliferative effect in HeLa cells (Figure 2B). Specifically, HeLa cell proliferation was suppressed by 22.8% and 53.5% following treatment with 350 and 641.2 μM boric acid for 24 h, respectively (*p* < 0.001 and *p* < 0.0001 vs. control). Overall, these results demonstrate that boric acid inhibited cell viability and proliferation more effectively in HeLa cells than in HUF cells, suggesting that HeLa cells are more sensitive to boric acid treatment.

We subsequently conducted western blot analysis of G1 phase‐related proteins to validate the BrdU assay findings (Figure 2C). In HUF cells, treatment with 350 μM boric acid did not alter the expression levels of cyclin D1 and CDK4, though a slight reduction was observed at the 641.2 μM concentration. However, in HeLa cells, both 350 and 641.2 μM boric acid treatments resulted in a concentration‐dependent decrease in the cyclin D1 and CDK4 levels.

### 3.3 Boric Acid Triggered Nuclear Abnormalities in HeLa Cells

**FIGURE 3:**
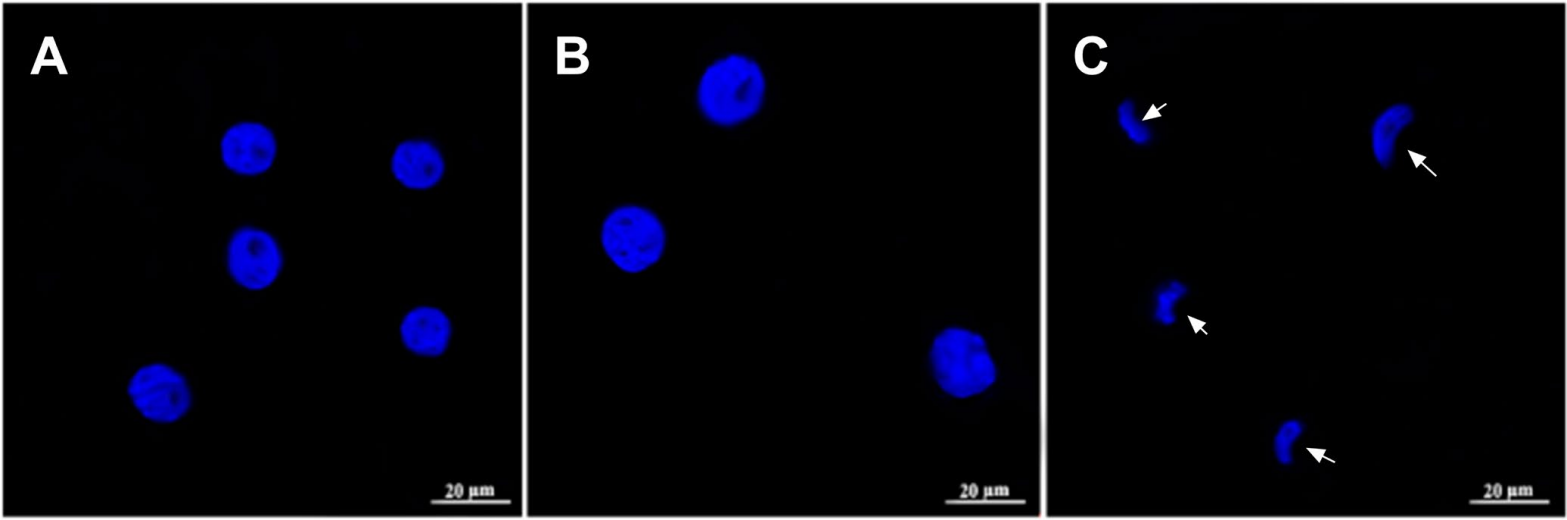
Effects of boric acid on nuclear anomalies in HeLa cells. (A‐C) DAPI staining of HeLa cells, (A) Control group, (B) 350 μM boric acid treatment group, (C) 641.2 μM boric acid treatment group. White arrows represent kidney‐shaped nucleus abnormalities.

The effects of boric acid on nuclear abnormalities in HeLa cells were observed following treatment with 350 and 641.2 μM boric acid for 24 h (Figure 3A–C). After 24 h of treatment with 350 μM boric acid, a reduction in HeLa cell numbers was noted compared to the control group, although there were no significant changes in nuclear morphology (Figure 3B). However, after 24 h of treatment with 641.2 μM boric acid, a pronounced decrease in cell numbers was observed, alongside a loss of normal cell morphology, with cells adopting a rounded shape (Figure 3C). The nuclei also exhibited irregular shapes, suggesting that boric acid may have disrupted the microfilament cytoskeleton in the cells.

### 3.4 Boric Acid Leads to Induction of Autophagosome Formation and Autophagic Markers in HeLa Cells

**FIGURE 4:**
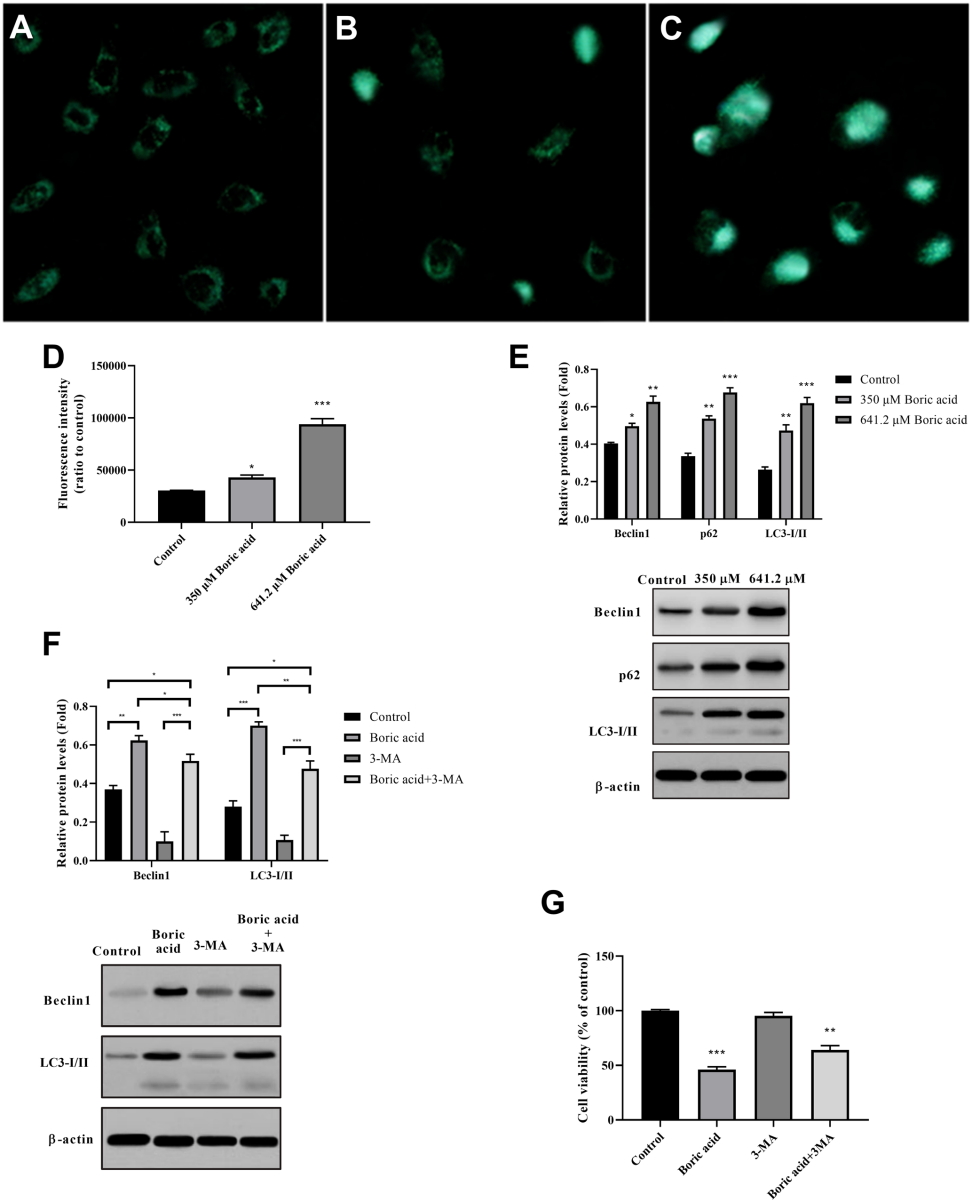
Boric acid treatment induced autophagy in HeLa cells. (A‐C) Green fluorescent particles in DC‐labelled HeLa cells following boric acid treatment indicate autophagosome formation, (A) Control group, (B) 350 μM boric acid treatment group, (C) 641.2 μM boric acid treatment group, (D) Fluorescence intensity varied with the degree of autophagy, (E) Beclin1, p62 and LC3‐I/II mRNA expressions in HeLa cells, (F) Analysis of boric acid, 3‐MA and boric acid+3‐MA treatment on Beclin1, p62 and LC3‐I/II mRNA expression in HeLa cells, (G) MTT results of boric acid, 3‐MA and boric acid+3‐MA treatment in HeLa cells. *p < 0.05, **p < 0.001 and ***p < 0.0001.

To investigate the effect of boric acid on autophagy, HeLa cells were exposed to 350 and 641.2 μM concentrations of boric acid for 24 h. Fluorescence microscopy revealed the aggregation of green fluorescent particles in cells treated with varying boric acid concentrations (Figure 4A–C). Treatment with 350 μM boric acid resulted in a partial increase in autophagosome formation (green fluorescent dots) as indicated by DC labelling, compared to the control group (Figure 4B). Moreover, treatment with 641.2 μM boric acid led to a pronounced induction of autophagosomes in HeLa cells (Figure 4C). Furthermore, as shown in Figure 4D, after 350 μM and 641.2 μM boric acid treatment, the fluorescence intensity associated with autophagosomes in HeLa cells was found to increase in a concentration‐dependent manner (*p* < 0.05 and *p* < 0.0001). These findings demonstrate that the number of autophagosomes in the cytoplasm of HeLa cells progressively increased with higher concentrations of boric acid.

Consistent with the autophagosome results, Western Blot analysis results showed a concentration‐dependent changes of Beclin1, LC3‐I/II and p62 protein levels in HeLa cells treated with 350 and 641.2 μM boric acid for 24 h (Figure 4E). 3‐MA is a well‐established autophagy inhibitor, known to block autophagosome formation. In this study, 3‐MA was employed to further explore the role of boric acid (641.2 μM) in autophagy regulation in HeLa cells. Notably, treatment with 1 mM 3‐MA for 24 h reduced the protein levels of Beclin1 and LC3‐I/II. In contrast, boric acid treatment for 24 h led to increased expression of Beclin1 and LC3‐I/II, indicating autophagy activation (Figure 4F). The data revealed that co‐treatment with boric acid and 3‐MA elevated the expression of Beclin1 and LC3‐I/II, while boric acid alone resulted in even higher levels. Moreover, MTT assay findings revealed a 53.7% reduction in cell viability in HeLa cells treated with 641.2 μM boric acid, whereas cells treated with the combination of 3‐MA and boric acid showed a 49.1% reduction in viability (Figure 4G). Collectively, these results confirm that boric acid plays a role in the induction of autophagy.

### 3.5 Boric Acid Activates ER Stress Through Autophagy and Apoptosis Pathways in HeLa Cells

**FIGURE 5:**
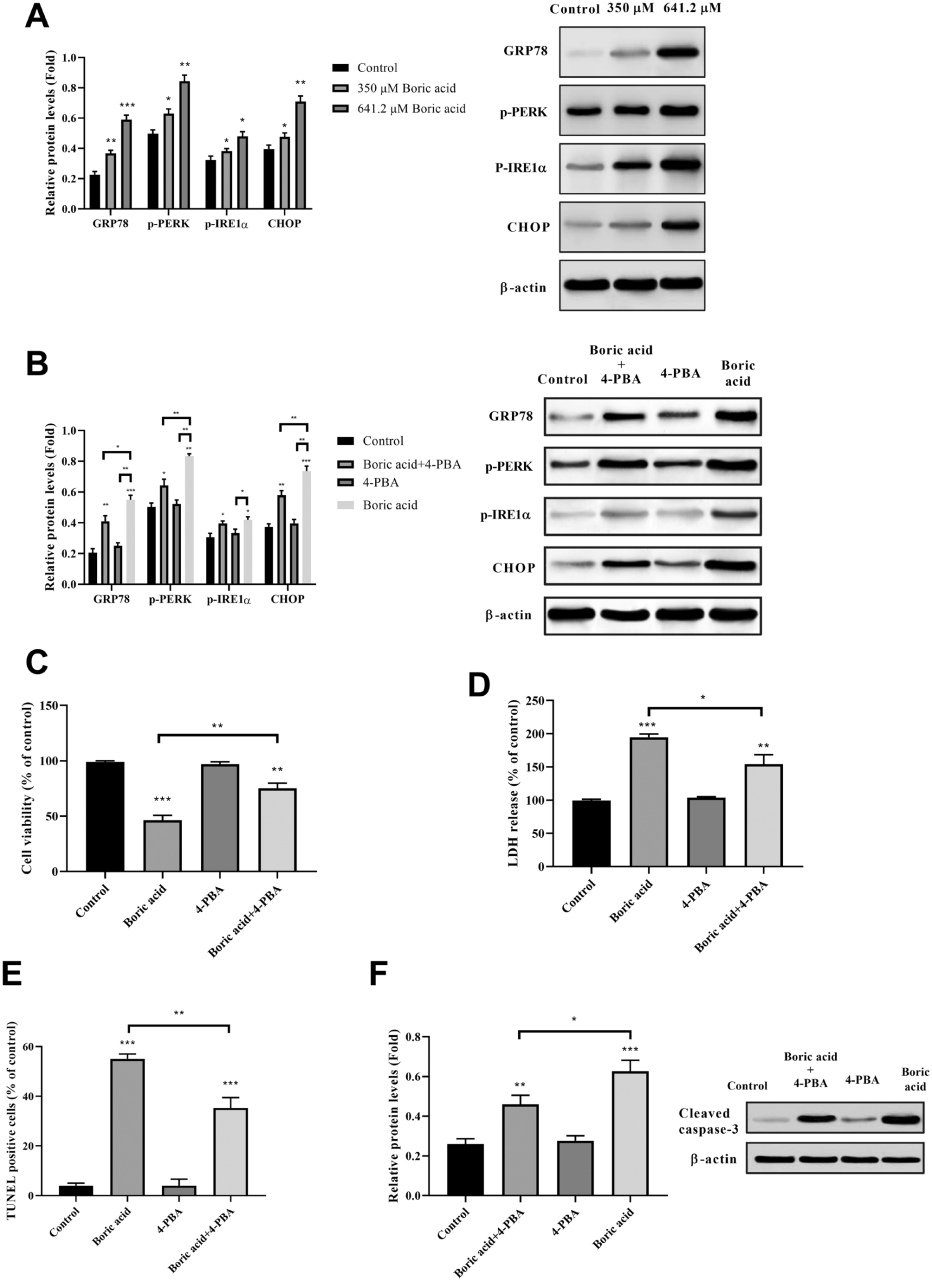
Boric acid treatment triggered ER stress in HeLa cells. (A) GRP78, p‐PERK, p‐IRE1α, and CHOP mRNA expression in boric acid‐treated HeLa cells, (B) Analysis of boric acid, 4‐PBA and boric acid+4‐PBA treatment on GRP78, p‐PERK, p‐IRE1α, and CHOP mRNA expression in HeLa cells, (C) MTT results of boric acid, 4‐PBA and boric acid+4‐PBA treatment in HeLa cells, (D) LDH release of boric acid, 4‐PBA and boric acid+4‐PBA treatment in HeLa cells, (E) TUNEL‐positive of boric acid, 4‐PBA and boric acid+4‐PBA treatment in HeLa cells, (F) Cleaved‐caspase 3 mRNA expression of boric acid, 4‐PBA and boric acid+4‐PBA treatment in HeLa cells. *p < 0.05, **p < 0.001 and ***p < 0.0001.

To assess whether boric acid activates ER stress in HeLa cells, we examined the expression of several ER stress markers, including GRP78, p‐PERK, p‐IRE1α and CHOP, via Western Blot analysis. We observed a concentration‐dependent increase in these markers when cells were treated with 350 and 641.2 μM boric acid, suggesting the induction of ER stress (Figure 5A). To determine if this ER stress contributed to apoptosis, HeLa cells were co‐treated with 641.2 μM boric acid and 1 mM 4‐PBA, an ER stress inhibitor, for 24 h. Figure 5B shows that 4‐PBA significantly attenuated the upregulation of ER stress markers caused by boric acid. Furthermore, a noticeable increase in cell viability was seen in the co‐treatment group (Figure 5C). LDH release assays also indicated that 4‐PBA alleviated the cytotoxic effects of boric acid (Figure 5D). In addition, the rate of apoptosis, quantified by the number of TUNEL‐positive cells, was reduced with the combined 4‐PBA and boric acid treatment (Figure 5E). Likewise, the levels of cleaved‐caspase 3 protein in HeLa cells increased following boric acid treatment but declined when treated with the combination of boric acid and 4‐PBA (Figure 5F). These results collectively suggest that ER stress is a contributor to boric acid‐induced apoptosis in HeLa cells.

**FIGURE 6:**
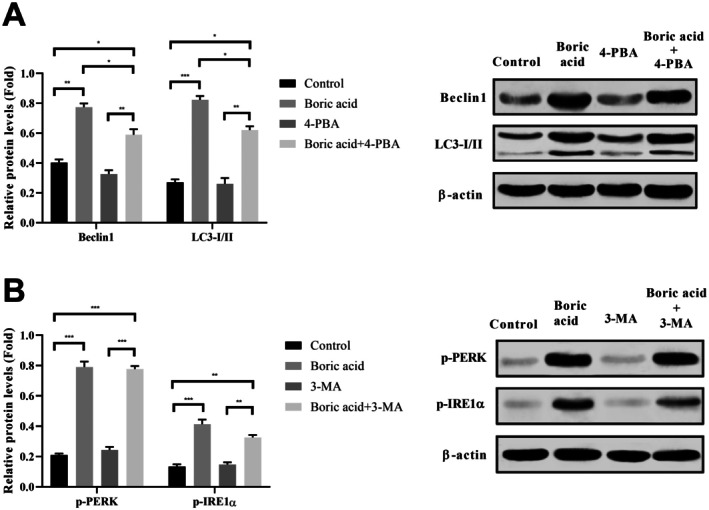
Effects of ER stress and autophagic inhibitors on Beclin1, LC3‐I/II, p‐PERK, and p‐IRE1α mRNA expressions in boric acid‐treated HeLa cells. (B) Analysis of boric acid, 4‐PBA, and boric acid+4‐PBA treatment on Beclin1 and LC3‐I/II mRNA expression in HeLa cells, (B) Analysis of boric acid, 4‐PBA, and boric acid+4‐PBA treatment on p‐PERK and p‐IRE1α mRNA expression in HeLa cells. *p < 0.05, **p < 0.001, and ***p < 0.0001.

To investigate whether autophagy activation serves as an adaptive response to ER stress, we analysed the LC3‐I/II and Beclin1 protein levels in HeLa cells following combined treatment with boric acid or 4‐PBA. As shown in Figure 6A, 4‐PBA effectively inhibited the boric acid‐induced upregulation of autophagy‐related proteins. In contrast, the use of the autophagy inhibitor 3‐MA did not significantly impact the expression of ER stress markers (p‐PERK and p‐IRE1α) in boric acid‐treated HeLa cells (Figure 6B). These findings suggest that boric acid‐induced ER stress is an upstream event that depends on autophagy.

## 4 Discussion

Research into new agents with anticancer properties is advancing to complement standard chemotherapy in current cancer treatments. Boron and its compounds stand out amongst these emerging agents. This study offers preliminary evidence of boric acid's potential anticancer effects in cervical cancer, demonstrating that boric acid effectively inhibits cervical cancer cell proliferation and induces ER stress through autophagy and apoptosis via caspase‐dependent pathways. Additionally, we observed that boric acid exposure leads to ER stress and heightened autophagosome formation in HeLa cells. Based on our results, we suggest that these anticancer effects may stem from the activation of ER stress and autophagy in cervical cancer cells. To our knowledge, our findings reveal a novel ER stress‐ and autophagy‐dependent pathway by which boric acid reduces cervical cancer cell viability, offering potential alternative therapeutic strategies for cervical cancer.

Our MTT assay results revealed an IC50 of 3.17 mM for boric acid in HUF cells, while in HeLa cells, the IC50 was significantly lower at 641.2 μM, indicating a more substantial reduction in cell viability in HeLa cells at lower concentrations. Our prior research demonstrated that borax, another boron‐based compound, displayed cytotoxicity at elevated concentrations (IC50 40.8 mM) in HL‐7702 cells but suppressed viability effectively at reduced concentrations (IC50 22.6 mM) in HepG2 cells [28]. In addition, in vitro experiments using various cell lines, including Ishikawa cells, highlighted a notable decrease in cell proliferation at boric acid levels of 40 mM and above [32]. Previous findings indicated that treatment with 10.77 mM boric acid significantly inhibited growth in DU‐145 cells [24]. Another investigation assessed the in vitro cytotoxic effects and potential anticancer properties of boric acid by examining colony formation, migration, invasion, cell proliferation, cell cycle progression, apoptosis, miRNA expression, and oxidative stress in the human ovarian cancer cell line MDAH‐2774 [33]. Collectively, existing literature underscores a pattern in which boron compounds hinder cancer cell proliferation at specific concentration thresholds. Boron Neutron Capture Therapy (BNCT) represents a targeted radiotherapy approach using tumour‐specific boron drugs such as boronophenylalanine (BPA) and sodium borocaptate (BSH), facilitating tumour‐specific damage while sparing adjacent normal tissues. Terada and colleagues reported that cervical cancer cells swiftly took up boron across three cervical squamous cell lines (SKG‐II, SiHa and C–4I) and HeLa, with uptake positively correlated with BPA concentration [34]. BNCT has demonstrated effectiveness against cervical squamous cell carcinoma and adenocarcinoma cells. Previous studies suggest that BNCT reduces cell proliferation, disrupts the cell cycle, compromises DNA and chromosomal integrity, and leads to pronounced transcriptomic changes in radiotherapy‐resistant cancer cells [35]. Consistent with these findings, our microscopic observations identified distinct morphological and nuclear abnormalities in HeLa cells following boric acid treatment.

Although our findings demonstrate significant cytotoxic and pro‐apoptotic effects of boric acid in HeLa cells with an IC50 of approximately 641 μM, we must critically assess the translational potential of these findings by evaluating whether such concentrations are achievable and clinically tolerable in vivo. Compared to conventional chemotherapeutic agents with lower micromolar or even nanomolar IC50 values, boric acid exhibits relatively moderate potency in vitro. However, clinicians must weigh the potential use of boric acid as a therapeutic agent against its safety profile and pharmacokinetic properties. Studies have shown that boron‐containing compounds, including boric acid and its derivatives, generally possess low acute toxicity in mammals. A previous study reported that oral administration of boric acid at doses up to 17.5 mg/kg/day in humans is within the acceptable daily intake (ADI), with chronic rodent studies identifying a no observed adverse effect level (NOAEL) at 9.6 mg/kg/day [36]. Notably, researchers have documented systemic administration of boric acid at doses achieving plasma concentrations above 1 mM in rodent models without causing severe toxicity, suggesting that concentrations in the range of our observed IC50 may be attainable under controlled conditions [37]. Additionally, pharmacokinetic data indicate that boric acid is rapidly absorbed after oral administration and distributes widely, primarily accumulating in soft tissues such as the liver, kidneys, and reproductive organs [38]. Its elimination is mainly renal, with a plasma half‐life of approximately 21 h in humans. Although achieving 600–700 μM plasma concentrations systemically would require dosing near or above current NOAEL levels, localised delivery systems (e.g., intratumoral injection, nanoparticle carriers or BNCT frameworks) may allow higher tumour‐selective concentrations with minimal systemic exposure. Therefore, while systemic administration of boric acid at cytotoxic levels observed in vitro may raise safety concerns, emerging delivery technologies and the favourable safety margins of boron compounds suggest that boric acid could hold promise as an adjunct or targeted therapeutic agent. Further preclinical studies are necessary to define the maximum tolerable dose, bioavailability, and tumour‐selective accumulation of boric acid in vivo.

Cell division is stringently regulated by a series of positive and negative control mechanisms, ensuring the accurate transmission of genetic material to future generations. CDKs and cyclins play a central role in this regulation, as the formation of CDK‐cyclin complexes activates CDKs as kinases, a process crucial for cell cycle progression [39]. In A2780 ovarian cancer cells treated with 2‐fluoro‐6‐formylphenylboronic acid, the downregulation of cyclin A‐CDK1/CDK2 and cyclin E‐CDK2 complexes (which manage the S phase) triggered S phase arrest, displaying antiproliferative effects on these cells [40]. Contrastingly, Cabus et al. reported no significant alteration in mRNA expression levels of Cyclin D1, Cyclin D2, CDK6 and CDK4 genes in MDAH‐2774 ovarian cancer cells exposed to boric acid compared to controls [33]. This finding suggests that apoptosis was induced through the mitochondrial pathway rather than by affecting cell cycle arrest‐related genes. In our investigation, we examined the effects of boric acid on cyclin D1 and CDK4 in HUF and HeLa cells. Notably, boric acid treatment at 350 and 641.2 μM concentrations led to no significant changes in cyclin D1 and CDK4 mRNA expressions in HUF cells, whereas we observed a concentration‐dependent reduction in HeLa cells. Furthermore, boric acid impacted cell cycle arrest‐associated genes in HeLa cells but showed no effect in primary uterine fibroblast cells. Consistent with DAPI staining, MTT, and BrdU assays (which assess nuclear integrity and cell viability), boric acid treatment induced nuclear abnormalities including vacuolization, fragmentation, and condensed nuclei specifically in HeLa cells.

Cervical cancer remains a significant global health challenge, ranking as the fourth most common cancer with approximately 600,000 new cases and 300,000 fatalities annually [3]. Despite advancements in surgical procedures and systemic radiotherapy and chemotherapy, the prognosis for women with cervical cancer often remains poor. This underscores the urgent need for research into chemotherapeutic resistance mechanisms and the development of alternative therapeutic approaches. Targeting the ER stress pathway has emerged as a potential avenue for novel cancer treatment, as prolonged ER stress can lead to cell death [41]. The ER is a crucial organelle within eukaryotic cells, involved in protein processing, intracellular calcium storage, and essential biosynthetic processes [42]. When protein folding capacity is overwhelmed, unfolded proteins accumulate within the ER lumen, causing ER stress. In cancer cells, ER stress responses aim to mitigate damage [43]. However, the specific signalling mechanisms of ER stress in boric acid‐treated HeLa cells remain largely unexplored. Key ER stress components like GRP78 and CHOP play pivotal roles in activating the stress signalling cascade [44]. Under normal conditions, GRP78 binds to transmembrane proteins (PERK, IRE1 and ATF6) within the ER lumen. During ER stress, GRP78 dissociates from these proteins, initiating stress signalling pathways. Additionally, CHOP functions pro‐apoptotically in stress responses [45]. Previous research has shown that boric acid enhances eIF2α phosphorylation and BiP/GRP78 translation in DU‐145 cells, indicating ER stress induction via eIF2α/ATF pathway activation [46]. In contrast, Xiong and colleagues observed that boric acid mitigated heat‐induced ER stress in mouse granulosa cells, evidenced by increased GRP78 and CHOP protein levels [47]. Our previous study determined that boric acid triggered ER stress by regulating GRP78, ATF4 and CHOP in U251 glioblastoma cells [48]. To examine ER stress mechanisms in HeLa cells, we analysed GRP78, p‐PERK, p‐IRE1α and CHOP expression. Our results indicate that boric acid upregulated these markers, inducing ER stress. Interestingly, boric acid attenuated heat stress‐induced increases in GRP78 and CHOP, suggesting it may reduce heat stress‐induced cell death by inhibiting ER stress in granulosa cells. To confirm ER stress induction, we applied ER stress inhibitor 4‐PBA to HeLa cells. As hypothesised, 4‐PBA suppressed boric acid‐induced ER stress. Furthermore, we measured cleaved caspase‐3 mRNA after 4‐PBA treatment and found reduced expression, verifying that boric acid‐induced ER stress contributes to apoptosis.

**FIGURE 7:**
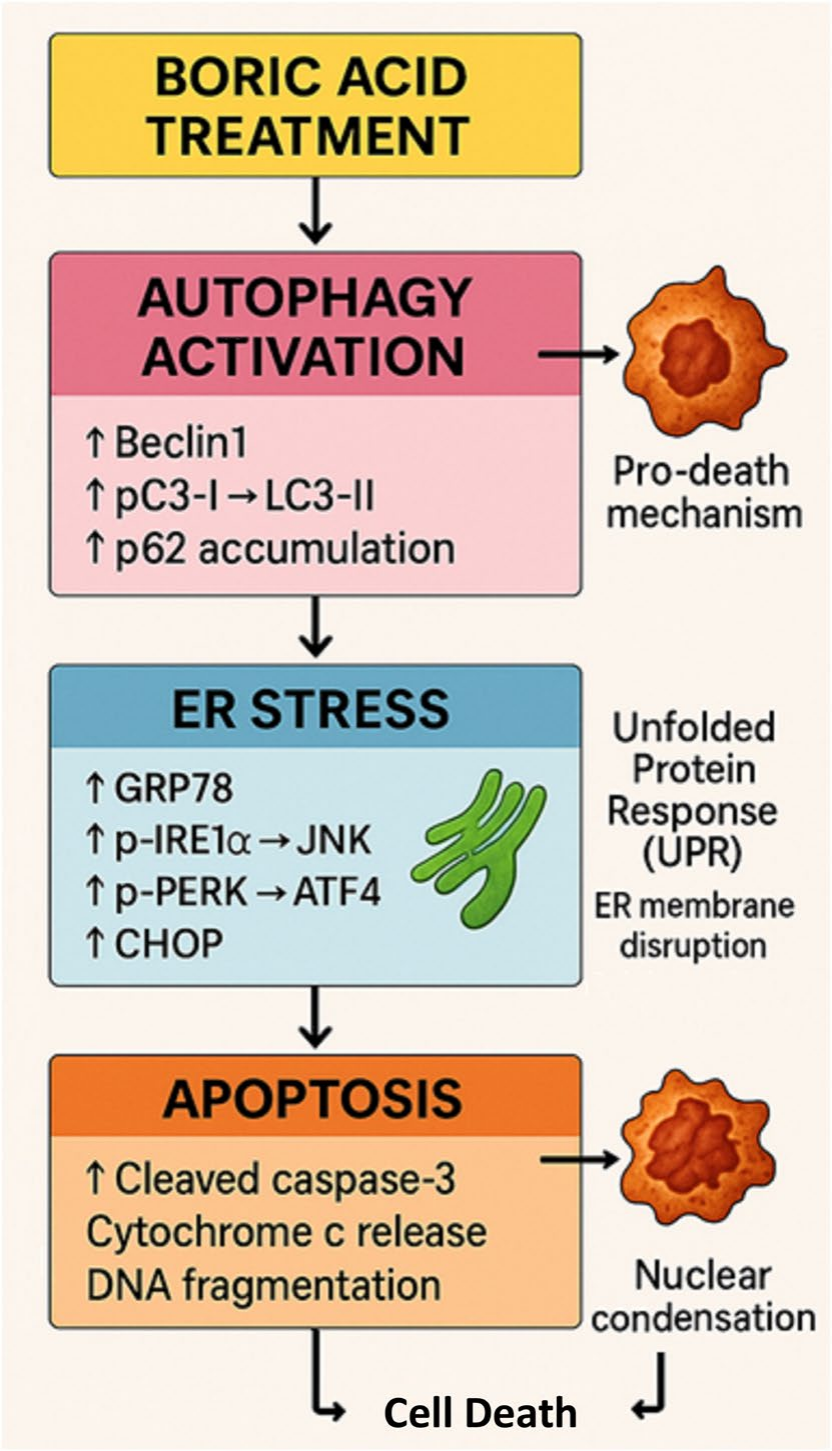
Schematic model of boric acid‐induced cytotoxic mechanisms via autophagy–ER stress–apoptosis axis in HeLa Cells.

Autophagy is a catabolic process activated by stress and cellular signalling, functioning as a homeostatic mechanism to degrade damaged organelles and misfolded proteins via lysosomes [49]. It serves as a critical recycling system within the cell. LC3, a soluble protein present in various cellular compartments, plays a key role in this process. During autophagy, the cytosolic form of LC3 (LC3‐I) converts to LC3‐II, a phosphatidylethanolamine‐conjugated form, which then incorporates into autophagosomal membranes [50]. The conversion of LC3‐I to LC3‐II, along with LC3 localisation in autophagosomes, serves as a hallmark of autophagy. Additionally, p62 interacts with LC3 to facilitate autophagosome formation. These autophagosomes fuse with lysosomes and undergo degradation, making p62 levels a potential indicator of lysosomal activity [51]. Treatment of HeLa cells with boric acid elicited a dose‐responsive elevation in the protein levels of both LC3‐II and p62. While this observation suggests autophagy pathway engagement, researchers must recognise that concurrent accumulation of LC3‐II and p62 does not provide definitive evidence for augmented autophagic flux. Such a biomarker profile is inherently ambiguous, as it can arise either from heightened autophagosome biogenesis or from compromised autophagosome degradation and turnover [52]. The LC3‐II protein, a standard marker for autophagosomal membranes, accumulates under conditions of both autophagy induction and lysosomal impairment [53]. Similarly, p62 (SQSTM1), an adaptor protein degraded via the autophagy‐lysosome pathway, typically diminishes during functional autophagic flux. The co‐increase of both LC3‐II and p62 observed in this study raises the possibility that boric acid may not only stimulate autophagosome formation but also potentially disrupt downstream processes, such as autophagosome‐lysosome fusion or lysosomal proteolytic activity. This interpretation aligns with established literature indicating that cellular stressors, including severe ER stress or reactive oxygen species (ROS), can impair lysosomal function, thereby causing autophagy blockade at the degradation stage [54]. In the absence of direct flux measurements—such as tandem fluorescent mRFP‐GFP‐LC3 assays, pharmacological inhibition with agents like Bafilomycin A1, or assessments of lysosomal activity—the functional outcome of boric acid on autophagy machinery in our experimental system remains indeterminate. Subsequent investigations should prioritise implementing these assays to differentiate between enhanced autophagy initiation and defective flux completion. Resolving this distinction is paramount, given that impaired autophagic flux frequently links to apoptotic cell death. This mechanistic insight could elucidate the basis for the concurrent increase in cleaved caspase‐3 detected in boric acid‐exposed cells. Beclin 1 is another crucial autophagy regulator, involved in degrading and recycling cellular components [55]. It exhibits tumour‐suppressive properties across various cancer cell types and regulates autophagic cell death by suppressing cell growth [56]. While autophagy generally serves to protect cancer cells from stress and sustain cellular homeostasis, excessive autophagy can lead to cell death, as demonstrated in recent studies. For instance, hexagonal boron nitride induces autophagy by promoting LC3 and lysosomal‐associated membrane protein 1 (LAMP‐1) autophagolysosome formation in human alveolar lung epithelial cells [57]. However, autophagy's role in boric acid‐treated cervical cancer cells remains unclear. Our findings indicate that boric acid induces both autophagy and ER stress in HeLa cells, leading ultimately to apoptotic cell death. The observed upregulation of autophagy markers—Beclin1, LC3‐I/II and p62—along with enhanced autophagosome formation, preceded significant increases in ER stress‐related proteins, including GRP78, p‐PERK, p‐IRE1α and CHOP. Furthermore, the concomitant elevation of cleaved‐caspase‐3 and increased apoptotic nuclei suggest a terminal cellular response. While autophagy is traditionally viewed as a cytoprotective stress response, increasing evidence suggests it can promote cell death under certain conditions, either independently or by amplifying ER stress‐induced apoptosis. In our study, the timing and correlation of increased autophagic activity with subsequent ER stress marker elevation and apoptosis imply that autophagy may act as an upstream pro‐death signal rather than a protective response (Figure 7). This interpretation aligns with prior studies reporting that sustained or excessive autophagy can shift from homeostatic to cytotoxic function, especially when ER homeostasis is irreversibly disrupted [58, 59]. Moreover, p62 accumulation—normally degraded during autophagic flux—suggests autophagy imbalance or blockade, which could contribute to cellular stress and apoptotic signalling. This aligns with previous tumour model observations where incomplete autophagy exacerbates ER stress and sensitises cells to apoptosis [60]. We observed that boric acid treatment upregulated Beclin 1, LC3‐I/II and p62 mRNA expressions in HeLa cells. Furthermore, boric acid increased both autophagosome number and fluorescence intensity (Figure 4), indicating autophagy induction. A critical question was whether this autophagy acted protectively or facilitated cell death. Our findings demonstrated that boric acid‐induced autophagy enhanced HeLa cell cytotoxicity. Interestingly, while boric acid +3‐MA (autophagy inhibitor) co‐treatment reduced HeLa cell viability versus 3‐MA alone and controls, boric acid alone significantly inhibited viability. These results suggest that boric acid promotes autophagic cell death. Furthermore, based on inhibitor studies (4‐PBA for ER stress; 3‐MA for autophagy), we propose that boric acid influences autophagy expansion by activating Beclin 1 and LC3 pathways via p‐PERK and p‐IRE1 activation.

To the best of our knowledge, no prior studies have investigated boric acid's effects on ER stress and autophagy in cervical cancer cells. Therefore, understanding ER stress and autophagy signalling roles in boric acid's antitumour activity is significant. In this study, we demonstrated that boric acid induces an ER stress response in HeLa cells, closely coordinated with autophagy induction. Moreover, boric acid modulated ER stress and autophagy‐related protein mRNA expression, thereby influencing cell cycle regulation, apoptosis, and proliferation. Although boric acid has demonstrated potential anticancer properties, its precise molecular targets in specific cancers remain largely undefined. Our findings provide a comprehensive molecular mechanism explanation for boric acid's modulation of ER stress and autophagy in cervical cancer. However, given boric acid's distinct effects on HUF versus HeLa, future research should focus on exploring whether boric acid can mitigate early cancer development in vivo using diverse models.

## Author Contributions

**Betul Keyif:** conceptualization (equal), data curation (equal), formal analysis (equal), resources (equal), visualization (equal). **Ceyhan Hacioglu:** conceptualization (equal), data curation (equal), formal analysis (equal), funding acquisition (equal), investigation (equal), methodology (equal), project administration (equal), resources (equal), software (equal), supervision (equal), validation (equal), visualization (equal), writing – original draft (equal), writing – review and editing (equal).

## Conflicts of Interest

The authors declare no conflicts of interest.

## Data Availability

The data that support the findings of this study are available from the corresponding author upon reasonable request.
